# Nanostructured La_0.75_Sr_0.25_Cr_0.5_Mn_0.5_O_3_–Ce_0.8_Sm_0.2_O_2_ Heterointerfaces as All-Ceramic Functional Layers for Solid Oxide Fuel Cell Applications

**DOI:** 10.1021/acsami.2c14044

**Published:** 2022-09-07

**Authors:** Juan de Dios Sirvent, Albert Carmona, Laetitia Rapenne, Francesco Chiabrera, Alex Morata, Mónica Burriel, Federico Baiutti, Albert Tarancón

**Affiliations:** † Department of Advanced Materials for Energy, Catalonia Institute for Energy Research (IREC), Jardins de les Dones de Negre 1, Sant Adrià del Besòs, Barcelona 08930, Spain; ‡ Univ. Grenoble Alpes, CNRS, Grenoble INP, LMGP, 38000 Grenoble, France; § Department of Energy Conversion and Storage, Functional Oxides group, Technical University of Denmark, Fysikvej, 310, 233, 2800, Kgs. Lyngby, Denmark; ∥ Department of Materials Chemistry, National Institute of Chemistry, Hajdrihova 19, Ljubljana SI-1000, Slovenia; ⊥ ICREA, Passeig Lluís Companys 23, 08010 Barcelona, Spain

**Keywords:** thin films, hydrogen oxidation reaction, symmetric functional layers, solid oxide cells, nanocomposites

## Abstract

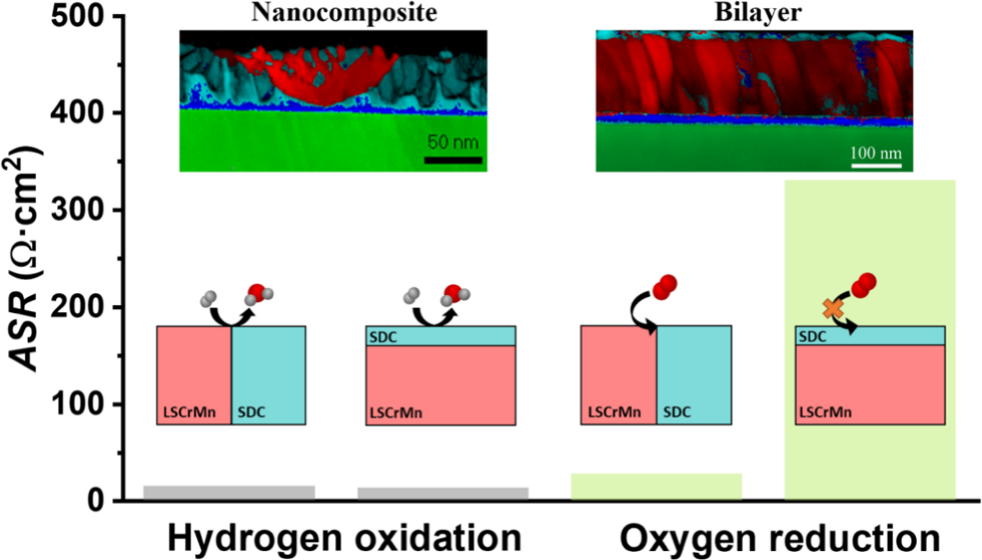

The use of nanostructured interfaces and advanced functional materials opens up a new playground in the field of solid oxide fuel cells. In this work, we present two all-ceramic thin-film heterostructures based on samarium-doped ceria and lanthanum strontium chromite manganite as promising functional layers for electrode application. The films were fabricated by pulsed laser deposition as bilayers or self-assembled intermixed nanocomposites. The microstructural characterization confirmed the formation of dense, well-differentiated, phases and highlighted the presence of strong cation intermixing in the case of the nanocomposite. The electrochemical properties—solid/gas reactivity and in-plane conductivity—are strongly improved for both heterostructures with respect to the single-phase constituents under anodic conditions (up to fivefold decrease of area-specific resistance and 3 orders of magnitude increase of in-plane conductivity with respect to reference single-phase materials). A remarkable electrochemical activity was also observed for the nanocomposite under an oxidizing atmosphere, with no significant decrease in performance after 400 h of thermal aging. This work shows how the implementation of nanostructuring strategies not only can be used to tune the properties of functional films but also results in a synergistic enhancement of the electrochemical performance, surpassing the parent materials and opening the field for the fabrication of high-performance nanostructured functional layers for application in solid oxide fuel cells and symmetric systems.

## 1 Introduction

In the recent years, there has been an increasing interest in the development of solid oxide cells (SOCs)^1^ and microsolid oxide cells (μ-SOCs)^2−4^ as promising alternative solutions to fossil fuel-based engines and portable batteries, respectively. SOCs present the advantage of being able to either produce electrical power coming from an electrochemical reaction (fuel cell mode) or store energy in chemical form (electrolysis mode). When implemented as μ-SOCs, these devices could potentially supply power to the consumer electronics sector and information and communication technologies, as well as act as microenergy storers. Nonetheless, the application of SOCs and μ-SOCs in real devices is strongly limited by the need for reaching high operating temperatures and by the poor long-term stability of the materials employed. Thus, solutions for improving the performance of SOCs are being extensively investigated for all their components.^5,6^ Nanostructuring electrode materials through thin-film approaches, including surface decoration^7^ and fabricating complex structures like multilayers^8,9^ and vertically aligned nanostructures^10^ (VANs), are some of the proposed strategies to enhance the solid-gas reaction kinetics and cell stability. In particular, the development of new self-assembled thin-film nanocomposites is a promising strategy for engineering the electrode–electrolyte interface, arguably a key area during SOC operation. Here, high electrochemical activity, in-plane electronic percolation, and stability against cationic diffusion should be ensured. VANs offer a fully dense structure, favorable out-of-plane geometry for mass transport, and the possibility of taking advantage of interface nanoionic effects for tuning the local chemistry.^11−13^ In VANs, the intimate alternation of the single-phase materials at the nanoscale allows to fully exploit strategies of electrode fabrication based on the maximization of the triple-phase boundary (TPB) density.^10,14^ Perovskite–fluorite-based VANs have already been explored as novel functional layers in SOFCs. Early studies carried out by Yoon et al.^15^ introduced the use of La_1–*x*_Sr_*x*_CoO_3_-Ce_1-*x*_Gd_*x*_O_2_ (LSC-CGO) VANs at the electrolyte–electrode interface. The application of such heterostructures increased the TPB density, leading to reduced polarization resistance. Ce_1-*x*_Gd_*x*_O_2_-(ZrO_2_)_*x*_:(Y_2_O_3_)_1-*x*_ (GDC-YSZ) VANs were later proposed by Su et al. for boosting the ionic conductivity of electrolytes in SOFCs, resulting in a significant increase of power output.^16^ Develos-Bagarinao et al. have also recently reported high electrochemical performance on a system with a La_1–*x*_Sr_*x*_Co_*y*_Fe_1-*y*_O_3_-Ce_1-*x*_Gd_*x*_O_2_ (LSCF-GDC) VAN functional layer.^17^ Dense thin films also enhance cell stability by providing a barrier against cation migration. A La_1–*x*_Sr_*x*_MnO_3_-Ce_1-*x*_Sm_*x*_O_2_ (LSM–SDC) VAN cathode has been proven by our group to suppress dopant segregation on the film’s surface.^18^ Morales et al. have shown that, by implementing a thin-film ceria layer at the electrolyte/electrode interface, continuous SOFC operation over 4500 h can be achieved with low degradation and high power >1 W·cm^–2^ at 750 °C.^19^

While research on nanostructured air electrodes has advanced, investigation of thin-film architectures for anode application remains a less explored field. State-of-the-art anode materials consist mainly of porous layers of metallic phases^20^ and metal-electrolyte composites.^5,21,22^ Nevertheless, such structures present several limitations in terms of structural stability at high temperatures and sulfur poisoning.^23^ Moreover, their implementation in microdevices is hindered by the poor in-plane percolation of porous layers and by fabrication incompatibilities with typical thin-film deposition techniques. As a result, there is an increasing effort in developing novel all-oxide materials that may reach the high performance of metallic-based anodes while retaining long-term stability. State-of-the-art full ceramic fuel electrodes are based on acceptor-doped ceria.^24−26^ Despite doped ceria presents an excellent catalytic activity upon the hydrogen oxidation reaction (HOR) as well as a certain degree of mixed ionic–electronic conductivity in reducing conditions, it is characterized by limited electronic conductivity^27^ causing current percolation losses. This limitation makes the implementation of ceria-based, metal-free anodes deeply challenging. Potential alternatives consist of the use of novel anode formulations, such as double perovskites Sr_2_MMoO_6_ (M = Ni, Mg, Fe),^28−30^ Sr_2–*x*_La_*x*_CoMoO_6_,^31^ and SrFe_0.2_Co_0.4_Mo_0.4_O_3-δ_ (SFCM) perovskites.^32^ This is also the case of the perovskite family of doped chromites and, in particular, Sr-doped lanthanum chromite manganites (La_1–*x*_Sr_*x*_Cr_1–*y*_Mn_*y*_O_3_—LSCrMn),^33−36^ which presents high mixed ionic–electronic conductivity and excellent thermal stability in reducing conditions.^37^ While (La,Sr)CrO_3_ is a predominant hole conductor even in reducing atmospheres, a decrease of the B-site oxygen coordination and formation of oxygen vacancies are achieved by doping with multivalent transition elements (e.g., Mn).^35,36,38^ Mn-doped (La,Sr)CrO_3_ has therefore extensively been used in the past as an anode material due to the combination of high electronic and oxygen vacancy conductivity. Particularly, the equimolar Cr:Mn ratio has been shown to exhibit the best tradeoff between phase stability and electrochemical activity.^35,39,40^ Nonetheless, the electrochemical performance of LSCrMn-based systems as standalone anode materials is far behind that of ceria- and cermet-based anodes, so new chromite formulations and doping strategies have been explored.^41−46^ Advanced structures relying on the utilization of heterointerfaces and nanocomposites have also been proposed recently—after the seminal works by Chueh and Jung on ceria-based electrodes^24−26,47^—including La_0.2_Sr_0.7_TiO_3-δ_–Gd_0.1_Ce_0.9_O_1.95-δ_ composites^48^ and a Ni-CGO cermet with extremely low content of Ni,^49^ achieving remarkable HOR activity. Furthermore, LSCrMn/ceria-based composites have been proposed as SOFC anodes in the past,^50,51^ combining the electrical properties and stability of LSCrMn with the superior electrochemical properties of doped ceria electrodes with respect to HOR. Such promising results demonstrate the potential of using LSCrMn–SDC advanced materials and heterointerfaces for functional layer application in SOC technologies.

In this work, we investigate the fundamental electrochemical properties of thin-film heterostructures having potential application as electrode functional layers, namely, an intermixed LSCrMn–SDC VAN-like nanocomposite, and a bilayer with a thin layer of SDC deposited on top of the LSCrMn film. Electrochemical impedance spectroscopy (EIS) is employed in symmetrical cells to analyze the enhancement derived from heterostructuring under both oxidizing and reducing conditions. The results reveal a higher electrochemical performance for both heterostructures in anodic conditions when compared to the single-phase constituting materials (in terms of HOR kinetics and in-plane conductivity). The nanocomposite also possesses remarkable and stable oxygen reduction reaction (ORR) kinetics in an oxidizing environment, for potential use as a functional layer in symmetric SOCs. These results demonstrate the possibility of obtaining highly active fuel functional layers by nanostructuring materials based on LSCrMn and SDC, as a result of a synergistic enhancement of the electrochemical properties at the heterointerfaces.

## 2 Results and Discussion

### 2.1 Thin Film Fabrication and Microstructural Characterization

**Figure 1:**
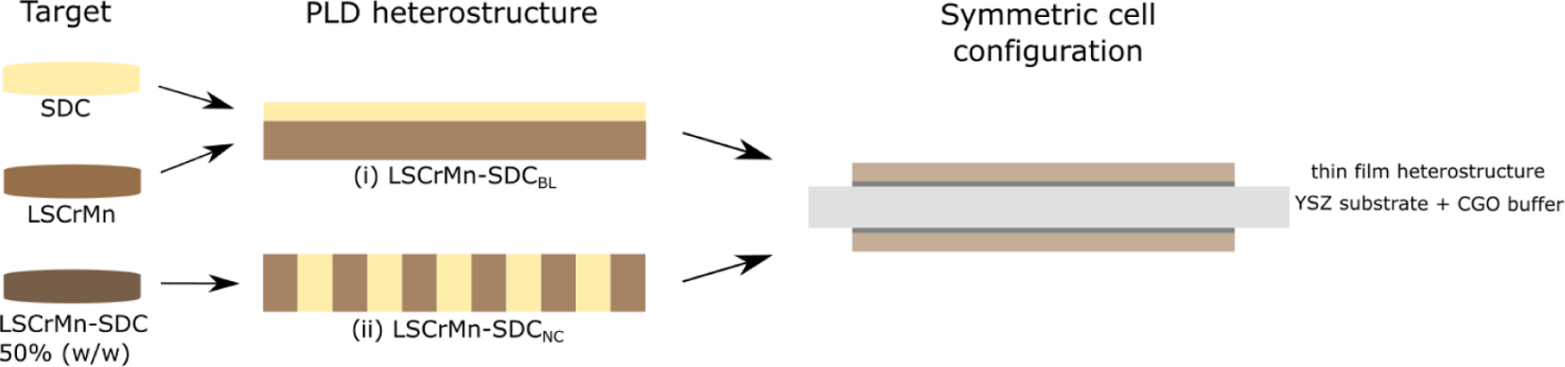
Schematic of the strategies followed for the deposition of the heterostructures: (i) bilayer obtained through a sequential two-step deposition of the parent materials and (ii) nanocomposite deposited from the ablation of a mixed-phase target.

Thin-film heterostructures and single-phase materials based on LSCrMn and SDC were deposited by pulsed laser deposition (PLD) on YSZ (100) single-crystal substrates. To prevent the formation of interface secondary phases, LSCrMn-containing films were grown with an additional ≈10 nm thick Ce_0.8_Gd_0.2_O_2_ buffer layer on top of YSZ.^52^ A sketch of the deposition strategies followed for the growth of the heterostructures is presented in Figure 1. The LSCrMn (thickness ≈150 nm)-SDC (≈8 nm) bilayer (from now on named LSCrMn–SDC_BL_) was obtained through sequential ablation of the LSCrMn and SDC single targets, whereas an LSCrMn–SDC intermixed target (50% w/w) was employed for the deposition of a nanocomposite (total thickness ≈135 nm) (LSCrMn–SDC_NC_). Please note that the use of a mixed target is typically employed for the fabrication of self-assembled VAN thin films.^18^ To rigorously evaluate the effect derived from heterostructuring, LSCrMn single-phase (150 nm) and SDC single-phase (50 nm) films were also fabricated and tested.

**Figure 2:**
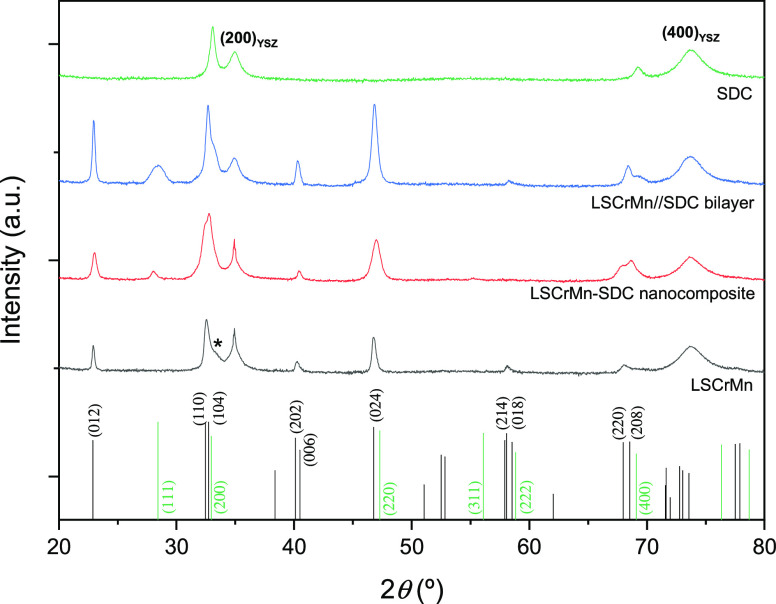
X-ray diffraction for all of the materials under study. Reference diffraction patterns are represented on the bottom for LSCrMn (*R*3̅*c* #167, in black)^53^ and SDC (*Fm*3̅*m* #225, in green),^54^ respectively. The most representative peaks are labeled. The asterisk (*) indicates a shoulder peak due to the presence of the (200) orientation of the CGO buffer layer. YSZ (*h*00) peaks are labeled directly on the SDC diffraction pattern as a common reference.

Information on the crystallographic properties of the different functional films can be derived from Figure 2, where we present the X-ray diffraction patterns measured for both heterostructures and the two single layers, compared to reference diffraction patterns for the two phases. LSCrMn shows in all cases a polycrystalline rhombohedral structure oriented along the (012), (110), and (104) planes, as expected from other studies on the LSCrMn system^41,53^ and confirmed by the TEM analysis performed (see later in the text). Single-phase SDC crystallizes in a cubic structure with preferential growth along the (100) orientation of the YSZ substrate. The two LSCrMn–SDC-based films present additional (111) and (220) orientations for the SDC phase. Please note that the (220) SDC peak falls close to the (024) orientation of LSCrMn, so the assignment may not be univocal. Slight peak asymmetries and position shifts are assigned to the overlapping and relative intensity of LSCrMn and SDC phases and to some cation interdiffusion in the case of the nanocomposite (cf. TEM later in the text). From this, we can conclude that phase differentiation between LSCrMn and SDC was obtained for all of the structures under consideration.

**Figure 3:**
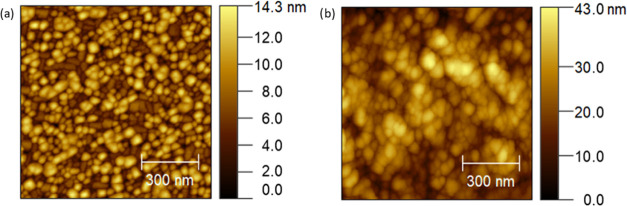
Top-view AFM micrographs of the heterostructures studied: LSCrMn–SDC_BL_ (2.4 nm *R*_ms_) (a) and LSCrMn–SDC_NC_ (5.5 nm *R*_ms_) (b).

Top-view atomic force microscopy (AFM) images of the heterostructures are shown in Figure 3. In Figure 3a, we report the micrograph of LSCrMn–SDC_BL_, while that of LSCrMn–SDC_NC_ is presented in Figure 3b. Both films present a dense microstructure with variations in roughness and grain size. The nanocomposite shows both higher roughness (*R*_ms_ = 5.5 nm) and grain size (≈30 nm) than the bilayer (*R*_ms_ = 2.4 nm and ≈20 nm grain size), most probably due to the more disordered, simultaneous growth of LSCrMn and SDC phases during the deposition.

**Figure 4:**
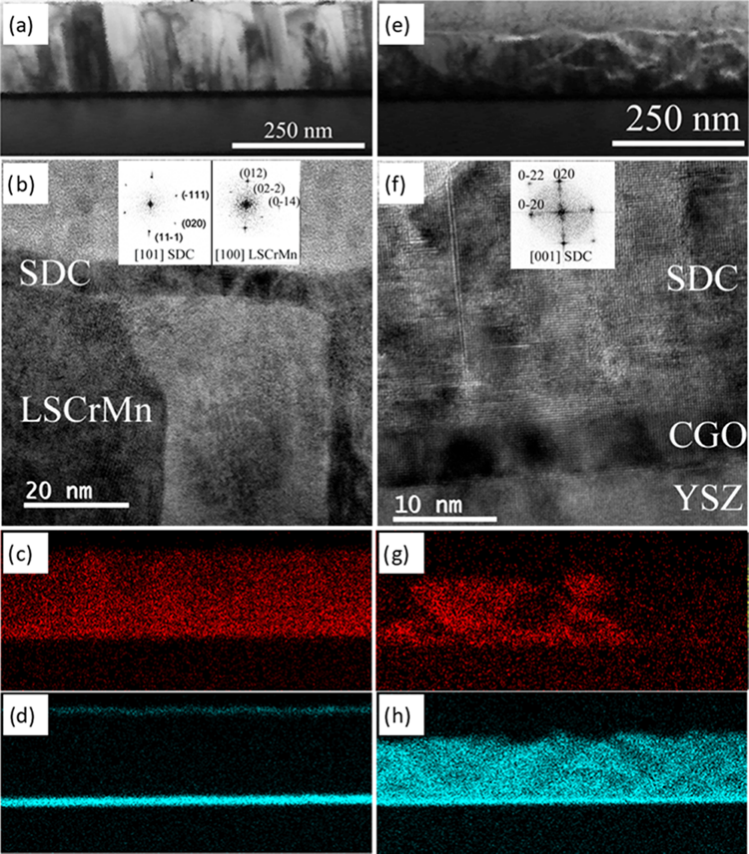
Cross-sectional characterization of the heterostructures studied: TEM and EDX mapping images (Sr–K signal in red, Ce–L in light blue) for LSCrMn–SDC_BL_ (a–d) and LSCrMn–SDC_NC_ (e–h). Insets in (b) and (f) show the related FT selected regions corresponding to each of the two phases.

Figure 4 presents the cross-sectional microstructural characterization performed on the two heterostructures by means of high-resolution transmission electron microscopy (TEM and HRTEM) and energy-dispersive X-ray spectroscopy (EDX) techniques. From low-magnification imaging, (panels (a) and (e) for the bilayer and nanocomposite, respectively), one can observe that all of the films analyzed are fully dense. HRTEM STEM is reported in Figure 4b (LSCrMn–SDC_BL_—related Fourier transform (FT) in the inset) and Figure 4f (SDC-rich area in LSCrMn–SDC_NC_—FT in the inset). Please refer to Supporting Figure S1 for the related selected area electron diffraction (SAED) pattern. For the bilayer, one can clearly observe the phase differentiation and morphology, with the perovskite phase growing in a columnar geometry (column width ≈30 nm), while the SDC deposited on top is fully crystalline and grows in a conformal continuous manner. Conversely for LSCrMn–SDC_NC_, by TEM imaging, one cannot resolve the phase alternation or a preferential growth structure in the nanocomposite layer. The STEM EDX mapping images for the LSCrMn–SDC_BL_ functional film, presented in Figure 4c (Sr), and d (Ce), confirm phase differentiation, with the majority of the Sr signal contained in the LSCrMn region and the Ce localized in the SDC and CGO top and bottom layers, respectively. A complete STEM EDX mapping image of the film with the contribution of more of the elements present is available in Figure S2. It must be noted that not all of the elements present in the films (e.g., Mn, Cr, Sm, and Gd) could be included in the analysis due to the overlapping of their EDX peaks. Hence, conclusions on cation interdiffusion and phase differentiation are based on the Sr and Ce signals reported. The STEM EDX analysis for LSCrMn–SDC_NC_ is reported in panels (g) and (h) for Sr and Ce, respectively. The film presents localized Sr-rich regions, which we assign to the localized perovskite phases (cf. also ASTAR later in the text). Nonetheless, Sr and especially Ce signals are present throughout the layers, suggesting strong cation intermixing during preparation (please note that this could also be partially assigned to grain overlapping in the cross-sectional observation). The complete EDX map of LSCrMn–SDC_NC_ is shown in Figure S3.

**Figure 5:**
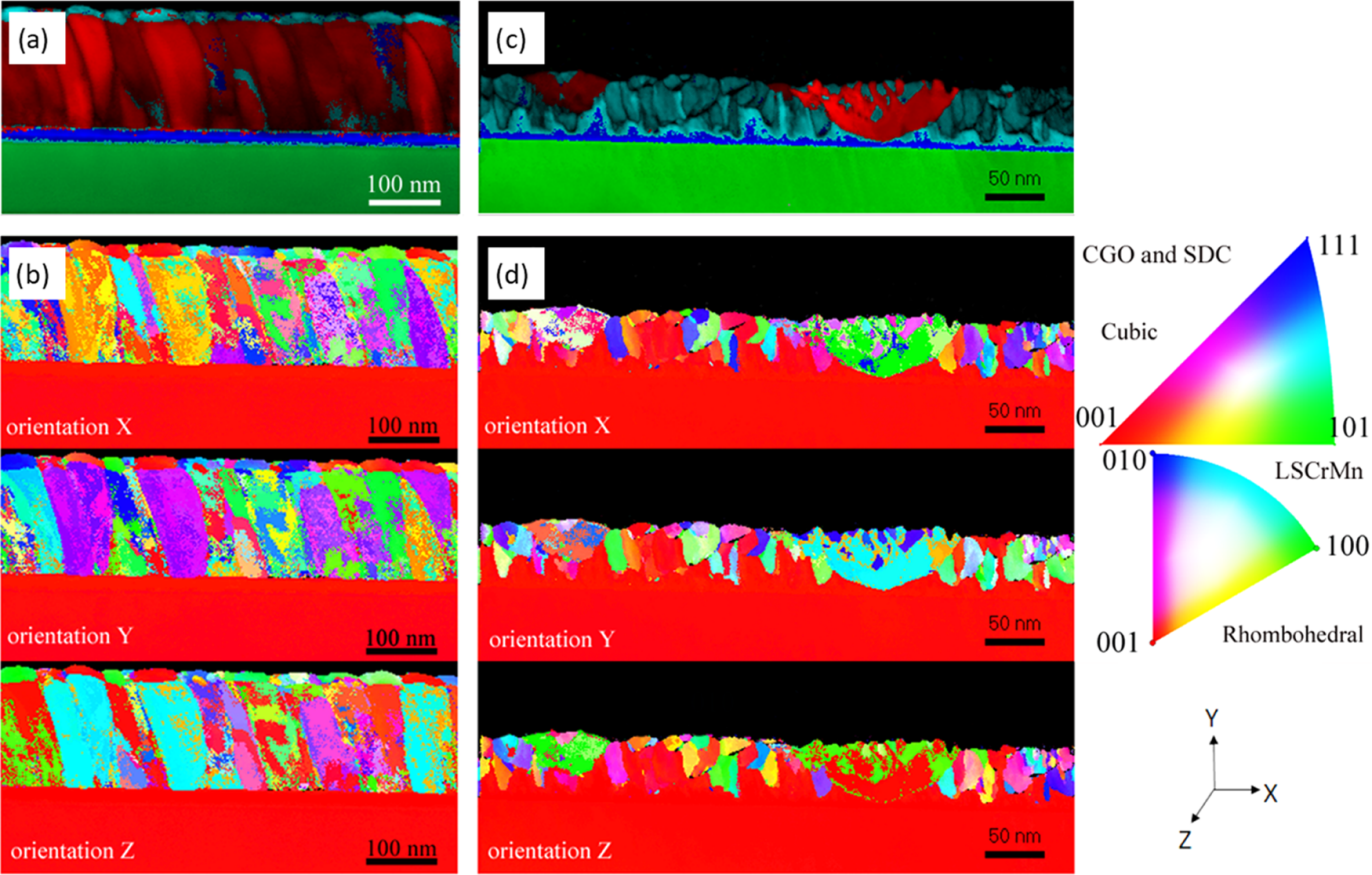
ASTAR phase and index analysis maps (top row) and corresponding orientation maps for each direction (bottom rows) of (a, b) LSCrMn–SDC_BL_ and (c, d) LSCrMn–SDC_NC_ films. Phase maps: YSZ in green, CGO in dark blue, LSCrMn in red, and SDC in light blue.

To improve the inherent spatial phase identification at the nanometer scale, we employed automated crystal phase and orientation mapping (ACOM) with a precession system (ASTAR), Figure 5. ASTAR analyses on LSCrMn–SDC_BL_ and LSCrMn–SDC_NC_ are shown in Figure 5, panels (a) and (c), respectively. This investigation confirms the presence of differentiated areas of rhombohedral phase (red, assigned to LSCrMn), cube-on-cube oriented *Fm*3*m* fluorite (dark blue, CGO buffer layer), and randomly oriented fluorite (light blue, SDC) for both heterostructures. In the case of LSCrMn–SDC_BL_, both the LSCrMn phase and the top SDC layer show polycrystalline growth, in agreement with the previous discussion on XRD. In relation to the map of the crystalline phases for LSCrMn–SDC_NC_ shown in panel (c), it is apparent that the nanocomposite layer is formed by a predominant cubic fluorite phase, whereas the rhombohedral LSCrMn is present as “boat-shape” isolated islands. Moreover, the occurrence of a preferential growth direction for the SDC cubic phase is observed in the orientation map in Figure 5d. Here, it can be seen that, for the first tens of nanometers, the SDC-rich phase grows preferentially following the same [100] direction as the CGO buffer. Figure S4 shows the overlapping of the electron diffraction pattern (experimental and theoretical) used for the identification of each phase.

We ascribe these findings to the more favorable growth of the ceria phase on the CGO-buffered YSZ substrate, owing to better lattice match and higher wettability, which causes the perovskite phase to grow in spatially segregated regions. Notably, according to ASTAR, this has not only an effect on the crystallographic order only but also on the phase fraction, as (highly intermixed) fluorite appears to be predominant over the rhombohedral perovskite. A likely explanation is that part of LSCrMn is dissolved in the fluorite matrix, as suggested by the observed high level of cationic intermixing (cf. EDX). It must be also pointed out that high levels of intermixing have been reported recently for a related perovskite–fluorite VAN according to dedicated atom probe tomography measurements.^18^

### 2.2 Electrochemical Performance

**Figure 6:**
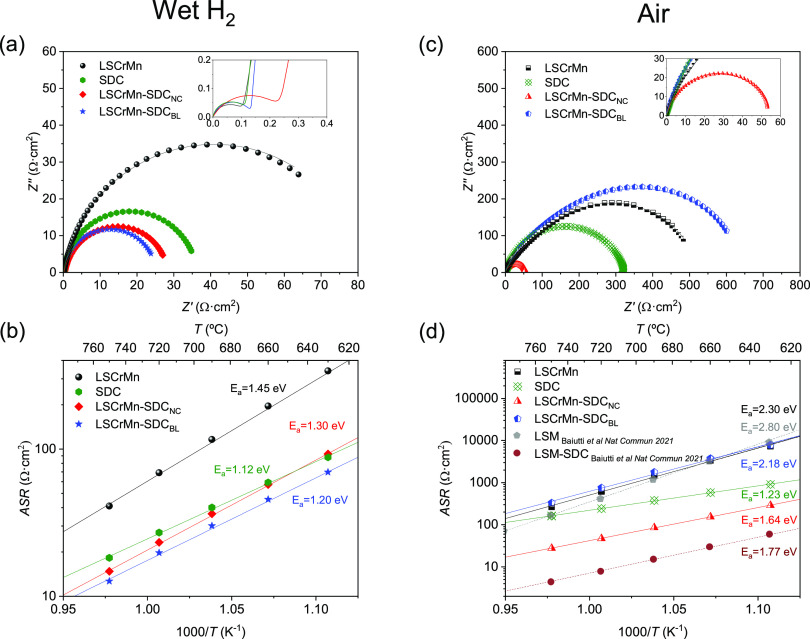
Impedance spectra for the materials under study (LSCrMn in black; SDC in green; LSCrMn–SDC_NC_ in red; and LSCrMn–SDC_BL_ in blue). Measurements performed under a wet hydrogen atmosphere: (a) Nyquist plots measured at 750 °C (dots) and the corresponding fitted arcs (solid lines) and (b) Arrhenius plot of the ASR. Measurements performed under air: (c) Nyquist plots measured at 750 °C (dots) and the corresponding fitted arcs (solid lines) and (d) Arrhenius plot of the ASR.

Figure 6a shows the Nyquist plot obtained by electrochemical impedance spectroscopy (EIS) at 750 °C for each material using symmetric cells (and porous gold current collectors) in a wet hydrogen atmosphere, along with the fitting obtained by modeling the corresponding equivalent circuit (continuous lines). All measurements present a main, well-defined arc at lower frequencies and a much smaller arc in the higher-frequency regime (fit spectra zoomed in the inset plot) (cf. also distribution of relaxation times (DRT) analysis in Supporting Figure S5). The obtained impedance spectra were fitted by modeling through the equivalent circuit shown in Figure S6a (simplified circuit originated from the more general Jamnik–Maier circuit, in Figure S6b)—note that fitting with the full Jamnik–Maier circuit was also tested and led to a similar analysis (not shown here for clarity reasons).^55^ According to this interpretation, the main arc is ascribed to surface oxidation reaction, whereas the high-frequency contribution could come from minor mass transport limitations (at the film/electrolyte interface or in the bulk material). This is consistent with studies done by Raj on oxygen diffusion and surface exchange studies on the LSCrMn system^39^ and by Primdahl,^56^ Jung,^24^ and Nakamura et al.^57^ A capacitance analysis is reported in Supporting Note 1. As expected, the LSCrMn single layer shows the highest area-specific resistance (*ASR* ≈ 41 Ω·cm^2^ at 750 °C—which takes into account the full polarization arc) among the materials tested,^40,58^ while single-phase SDC presents a lower polarization (≈18.2 Ω·cm^2^). Slightly lower *ASR* values at the reference *T* = 750 °C characterize LSCrMn–SDC_NC_ (≈14.7 Ω·cm^2^) and LSCrMn–SDC_BL_ (≈12.6 Ω·cm^2^). By analyzing the temperature dependence of *ASR* (Figure 6b), one can observe that the two functional heterostructures show the best performance in the full temperature range studied. The observed enhancement in the HOR kinetics with respect to single-phase LSCrMn and SDC suggests a synergistic effect of the two materials in the nanostructures. Very interestingly, such an improvement occurs irrespective of the type of heterostructure chosen, i.e., bilayer or nanocomposite. This is coherent with the microstructural analysis performed in Figures 4 and 5: In both cases and as inferred from the specific activity of the single phases (cf. Figure 6b), SDC provides reactive sites for fast HOR, whereas LSCrMn offers an electronic pathway. Thus, similar catalytic performances are expected owing to the exposed SDC–gas interface leading to an overall decrease in ASR, thanks to the enhanced electronic conductivity with respect to single-phase SDC.

Figure 6c,d shows the analysis of the electrochemical properties of our material systems under oxidizing conditions by out-of-plane EIS (Nyquist plot at 750 °C and Arrhenius plot of the ASR, respectively). This allows to further clarify the role of the constituting materials in the heterostructures and to evaluate their compatibility to be potentially employed in fully symmetric SOC devices. In air at high temperatures, one expects none of the components to be active if taken singularly, as LSCrMn presents predominant electronic conduction with negligible ionic conductivity,^40^ while SDC is a pure ionic conductor (cf. Figure 6d).^59,60^ Interestingly, the nanocomposite exhibits a remarkable performance also in air with comparable ASR (27.8 Ω·cm^2^), whereas much higher values are measured for all of the other systems considered (162.1, 264.7, 330.5 Ω·cm^2^ for single-phase SDC, LSrCrMn, and the bilayer, respectively, in oxidizing conditions) at 750 °C. A closer observation of the impedance spectra in Figure 6c highlights an evident depression of the arcs for LSCrMn and LSCrMn–SDC_BL_, arguably caused by the appearance of a Warburg-type impedance element in the higher-frequency range, indicating that polarization is colimited by diffusion and surface reactions and is well in line with the expected poor oxygen diffusivity of LSCrMn under oxidizing conditions (complete Jamnik–Maier equivalent circuit).^61,62^ In the cases of the bilayer configuration and single-phase SDC (the latter presenting an almost perfect impedance semicircle with fitting parameter *n* = 0.84^63,64^), the top ceria layer behaves as a pure ionic conductor,^59,60^ blocking the surface exchange. This analysis is supported by the DRT results collected in Figure S6b, which show the appearance of an additional high-frequency contribution (oxygen diffusion) for LSCrMn and LSCrMn–SDC_BL_ films. The DRT plot also shows a shift for the SDC peak toward the lower characteristic time region, confirming its behavior as a pure ionic conductor. Most importantly, the nanocomposite is characterized by faster ORR kinetics. Here, the SDC compensates for the lack of oxygen vacancies and ionic conductivity of the LSCrMn under cathodic conditions (i.e., SDC provides a fast mass transport pathway for out-of-plane for oxygen migration), while electronic conductivity (for the oxygen reduction reaction and for current collection) is provided by the perovskite phase. (Please note that a simple *RCP* element was employed for evaluating the *ASR* in the case of LSCrMn–SDC_NC_ (*n* = 0.86), confirming that surface exchange is limiting.)

Such a clear example of nanoengineered material—in which electronic and ionic conduction pathways are provided separately by the two phases—is achieved here despite the large disorder and cationic intermixing highlighted in Figures 4 and 5. The temperature dependence of the total *ASR* presented in Figure 6d reports, as a comparison, reference values for a traditional single-phase mixed ionic–electronic conductor (La_1*–x*_Sr_*x*_MnO_3_—LSM) and for a vertically aligned LSM–SDC nanocomposite, which was recently fabricated by our group.^18^ LSCrMn–SDC_NC_ surpasses the performance of single-phase LSM, and it is less than an order of magnitude higher than LSM–SDC in the lower-temperature regime, validating the thin-film nanocomposite approach for the fabrication of systems with *ad hoc* tailored performances. The surface exchange coefficient *k*_q_ values are summarized in Figure S7. In Supporting Note 2, we discuss the effect of thickness on the mass transport properties of the reported heterostructures.

**Figure 7:**
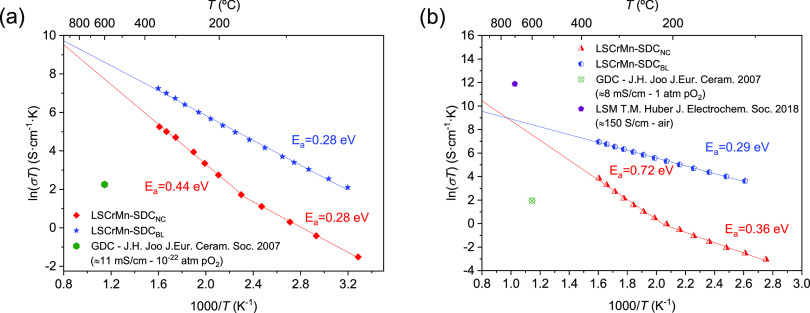
In-plane conductivity measured for the different heterostructures as a function of temperature under (a) 5% H_2_ in Ar and (b) synthetic air atmosphere.

The plot in Figure 7a shows sheet conductivity dependance on temperature in an Arrhenius-like representation for the two heterostructures measured under a 5% H_2_/Ar atmosphere. Both materials present linear behavior of ln(σT) vs 1/T, suggesting a small polaron hopping transport mechanism.^37,65^ In the case of LSCrMn–SDC_NC_, two linear regimes can be distinguished, most likely due to the presence of the two LSCrMn and SDC phases in the film. The activation energy extracted in the lower-temperature range (*E*_a_ = 0.28 eV) can be related to the LSCrMn phase as in the case of LSCrMn–SDC_BL_, which shows the same activation energy. In the higher-temperature range, the activation energy of LSCrMn–SDC_NC_ slightly deviates toward higher values (*E*_a_ = 0.44 eV) due to the SDC phase contribution at higher temperatures. Importantly, heterostructuring not only determines faster HOR (cf. Figure 6a,b) but also leads to the enhancement of more than three orders of magnitude in in-plane conductivity when compared to state-of-the-art doped ceria anode (σ ≈ 7.5 × 10^–4^ S·cm^–1^ at 500 °C)^27,60^—the bilayer being the most conductive owing to the continuous LSCrMn bottom layer (σ ≈ 2.2 vs 0.3 S·cm^–1^ for the bilayer and the nanocomposite at 350 °C, respectively).

We evaluated the sheet conductivity of LSCrMn–SDC_NC_ also in oxidizing atmospheres for validating the possible application as a functional layer in a symmetric cell configuration (Figure 7b), in comparison to that of LSCrMn–SDC_BL_. In the intermediate temperature regime, LSCrMn–SDC_NC_ is characterized by relatively low in-plane conductivity (σ ≈ 1.7 vs 0.08 S·cm^–1^ for LSCrMn–SDC_BL_ and LSCrMn–SDC_NC_ at 350 °C, respectively); i.e., the SDC phase prevents the formation of a percolating electronic pathway. Similar to the measurements performed under reducing conditions, sheet conductivities present a linear evolution with temperature and two differentiated linear regions for LSCrMn–SDC_NC_. Interestingly, owing to the large activation energy in the high-temperature regime (0.72 eV), the extrapolated conductivity value for LSCrMn–SDC_NC_ in SOFC operating conditions is σ ≈ 5.2 S·cm^–1^ at 700 °C. This is much higher than typically employed ceria-based functional thin-film layers^19^ (cf. Figure 7) and is closer to values for mixed ionic–electronic conductors for SOC applications such as LSM^66^ (notwithstanding the uncertainty deriving from extrapolation). Direct measurement of in-plane conductivity at high temperatures is prevented by the parallel contribution of the YSZ substrate.

**Figure 8:**
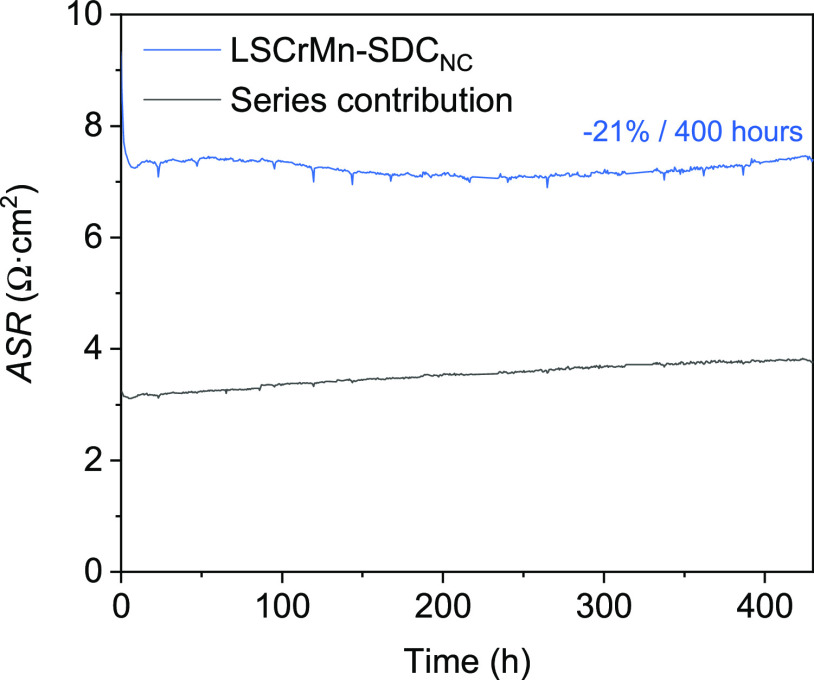
Degradation over time of the electrochemical performance of LSCrMn–SDC_NC_ at 780 °C in air: ASR contribution of LSCrMn–SDC_NC_ (blue) and the series contribution (black).

We report in Figure 8 a thermal degradation test performed for LSCrMn–SDC_NC_ at 780 °C during 400 h in OCV conditions (air atmosphere). The behavior of the film shows an initial improvement of the electrochemical performance (i.e., decrease of the associated resistance), going from an initial value of 9.32 to 7.14 Ω·cm^2^ after ≈200 h, and a very low degradation rate for the rest of the experiment (≈1.57%/100 h). Similar behavior on perovskite–fluorite composite systems has been previously attributed to the suppression of Sr segregation on the surface.^18,67^ This result unveils the potential of LSCrMn–SDC nanocomposites as stable functional layers against thermal degradation. We stress again here that the most relevant comparison is with fully dense structures that can potentially be employed as functional layers (in combination with a porous electrode): LSCrMn–SDC_NC_ outperforms in air the electrochemical activity of LSM (cf. Figure 6 and refs (18, 61)) and it is far more stable than state-of-the-art LSC.^68^ Moreover, both LSCrMn–SDC_NC_ and LSCrMn–SDC_BL_ present comparable performance to SDC in reducing conditions. With respect to traditional dense composites, the thin-film approach is able to provide intimate contact with the constituting phases at the nanoscale with no tortuosity, which translates into high electrochemical activity (cf. Figure 6) and good in-plane percolation (cf. Figure 7). The results also indicate LSCrMn–SDC_NC_ as a viable candidate for functional layers in the symmetric cell configuration (cf. Figure 6).^10,14,69,70^ Note that a further performance improvement is anticipated by improving phase alternation (cf. also previous work of the group in ref (18)), e.g., by changing the initial weight ratio in the PLD target.

## 3 Conclusions

The implementation of all-ceramic thin-film heterostructures as functional layers in solid oxide anodes and in symmetric SOCs is proposed in this work. LSCrMn–SDC thin films were fabricated by PLD as intermixed nanocomposites and bilayers and compared to the single-phase counterparts. The resulting microstructural characterization reveals the formation of dense films with clear phase differentiation and a large degree of cationic intermixing in the case of the nanocomposite. An enhancement of the electrochemical performance for the heterostructures with respect to single LSCrMn and SDC counterparts is highlighted under anodic conditions (area-specific resistance and in-plane conductivity), opening the possibility for using such thin-film-based heterointerfaces for catalyzing hydrogen oxidation in solid oxide cells. The LSCrMn–SDC nanocomposite is also characterized by remarkable performance in oxidizing conditions, as well as outstanding thermal stability for more than 400 h. The results obtained show how heterostructures take advantage of the functional properties of the parent compounds, giving rise to a synergistic effect between both phases and validating an approach based on thin-film self-assembly for the fabrication of high-performance nanocomposite functional layers in full symmetric SOC devices. For practical implementation in real devices—out of the scope of this work—, it would be of particular interest to combine the proposed functional nanocomposite with an extra porous layer. In such an architecture, an improvement in the electrochemical performance of the electrode would be indeed expected stemming from the increase in the active surface area.

## 4 Experimental Section

### Thin Film Deposition

SDC, LSCrMn, LSCrMn–SDC_NC_, and LSCrMn–SDC_BL_ thin films were deposited in a large-area pulsed laser deposition chamber (PVD Systems, PLD 5000) equipped with a 248 nm KrF excimer laser (Lambda Physics, COMPex PRO 205) on YSZ (100) substrate (Crystec GmbH). The deposition conditions are defined as follows: temperature 800 °C; oxygen pressure 0.007 mbar; target–substrate distance 90 mm; laser fluency ≈0.8 J·cm^–2^; laser frequency 2 Hz for LSCrMn–SDC_NC_ and 10 Hz for LSCrMn, SDC, and CGO single phases.

LSCrMn single-phase and LSCrMn–SDC_NC_ films were fabricated using homemade targets obtained by mixing commercial powders of La_0.75_Sr_0.25_Cr_0.5_Mn_0.5_O_3_ and Ce_0.8_Sm_0.2_O_2_ (1:1 wt %, Kceracell) via ball milling in ethanol solution. The dried powder mix was uniaxially pressed (7 MPa, 30 s) to form a pellet (diameter ≈ 1 inch). Sintering was carried out at 1300 °C for 4 h (heating and cooling ramps ≈5 °C/min). A commercial target was used for SDC and CGO.

### Thin Film Microstructural Characterization

Crystallographic information was retrieved by a Bruker D8 Advanced diffractometer equipped with a Cu Kα radiation source. XRD measurements were carried out in the θ–2θ configuration. Topographical microstructural characterization was performed in contact mode using an XE 100 model atomic force microscope provided by Park System Corp. TEM LSCrMn–SDC_BL_ and LSCrMn–SDC_NC_ specimens were prepared in cross section by the semiautomated polishing tripod technique with a MultiPrep system (Allied High Tech Products, Inc.). The PIPS II system from GATAN was used for final polishing. TEM and HRTEM images were recorded with a JEOL JEM 2010 LaB6 microscope operating at 200 kV with a 0.19 nm point-to-point resolution. EDX mapping was performed by STEM with a JEOL 2100F FEG microscope operating at 200 kV with a 0.2 nm resolution in the scanning mode and equipped with a JEOL SDD Centurio detector with a large solid angle of up to 0.98 sr. The local structural properties of LSCrMn–SDC_BL_ and LSCrMn–SDC_NC_ heterostructures were further investigated by automated crystal phase and orientation mapping (ACOM) with a precession system (ASTAR) implemented in the JEOL 2100F FEG microscope. The crystal phase and orientation maps were obtained by the precession of the primary electron beam around the microscope’s optical axis at an angle of 1.2° while collecting the electron diffraction patterns at a rate of 100 frames/s with a step size of 2 nm for LSCrMn–SDC_BL_ and 3 nm for the LSCrMn–SDC_NC_ sample. In this technique, the incident electron beam was a few nanometers in size and was processed to reduce the dynamical effects and to enhance the indexing quality. The electron beam was simultaneously scanned over the area of interest to record an electron diffraction pattern at each location. The experimental electron diffraction patterns were basically compared with the complete set of theoretical diffraction patterns, which were computed for every expected crystalline phase and for a large number of orientations. The best match between the experimental and theoretical electron diffraction patterns permitted the identification of both the crystalline phase and orientation with high precision.

### Electrochemical Characterization

The electrochemical characterization of the films was performed with a Novocontrol impedance spectrometer, with a frequency range of 1 MHz–0.1 Hz, open circuit potential, and an AC amplitude of 50 mV. The measurements were performed in a symmetric configuration with the films deposited on both sides of the CGO/YSZ substrates. Porous Au paste (Fuel Cell Materials) was brushed on top of the films to minimize any possible current percolation losses. The atmosphere set for the characterization in reducing conditions was 100 mL/min pure H_2_ passing through a bubbler, while the measurements in an oxidizing atmosphere were performed using 100 mL/min synthetic air. The measurement was carried out in a ProboStat station (NorECs) placed in a vertical furnace. The thermal aging experiment was carried out at 780 °C in a similar setup using a PARSTAT 2273 potentiostat with the same AC input signal and measuring impedance spectra every 30 min. The cell was mounted in an asymmetric configuration with the functional film deposited on one side of the CGO/YSZ substrate. The Ag paste (Sigma-Aldrich) was brushed on the opposite side to act as a low-impedance electrode. The rest of the setup remained the same as in the electrochemical characterization measurements.

### In-Plane Conductivity Measurements

For the in-plane conductivity measurements, the films were deposited on CGO/YSZ substrates for the LSCrMn–SDC-based thin films and on a sapphire (0001) single-crystal substrate for the LSCrMn single-phase films. The deposition conditions were kept the same as described previously for each film. The conductivity measurements were performed in a four-probe heating station (Linkam Instruments THMS600) using van der Pauw configuration. The Ag paste was brushed for improving the electrical contact between the film and the probe. The reducing atmosphere set was 5% H_2_ in Ar with a flow of 100 mL/min. The oxidizing atmosphere set was synthetic air at 100 mL/min. The temperature range was restricted to 350 °C to avoid any parasitic current from the YSZ substrate.

## Supporting Information Available

SAED patterns of the heterostructures fabricated, EDX elemental distribution maps, ASTAR phase identification of the nanocomposite, DRT analysis of the impedance spectra measured at 750 °C, equivalent circuits employed for fitting the impedance spectra, Arrhenius plot of the oxygen surface exchange coefficient, analysis of the capacitance extracted from the impedance measurements, and discussion note on the mass transport properties of the films studied (PDF)
