# Supplementary material for: Nanostructured La0.75Sr0.25Cr0.5Mn0.5O3–Ce0.8Sm0.2O2 Heterointerfaces as All-Ceramic Functional Layers for Solid Oxide Fuel Cell Applications
Source: ACS Appl Mater Interfaces. 2022 Sep 7;14(37):42178–87. doi: 10.1021/acsami.2c14044 (PMC9501924; doi:10.1021/acsami.2c14044)
Supplement: Supplementary file 1 — am2c14044_si_001.pdf [file am2c14044_si_001.pdf]

Nanostructured $La_{0.75}Sr_{0.25}Cr_{0.5}Mn_{0.5}O_3$-$Ce_{0.8}Sm_{0.2}O_2$ heterointerfaces as all-ceramic functional layers for solid oxide fuel cell applications

Juan de Dios Sirvent[a], Albert Carmona[a], Laetitia Rapenne[b], Francesco Chiabrera[a,c], Alex Morata[a], Mónica Burriel[b], Federico Baiutti[a,d], Albert Tarancón*[a,e].*

[a]Department of Advanced Materials for Energy, Catalonia Institute for Energy Research (IREC), Jardins de les Dones de Negre 1, Sant Adrià del Besòs, Barcelona 08930, Spain

[b]Univ. Grenoble Alpes, CNRS, Grenoble INP, LMGP, 38000 Grenoble, France

[c]Department of Energy Conversion and Storage, Functional Oxides group, Technical University of Denmark, Fysikvej, 310, 233 2800 Kgs. Lyngby, Denmark

[d]Department of Materials Chemistry, National Institute of Chemistry, Hajdrihova 19, Ljubljana SI-1000, Slovenia

[e]ICREA, Passeig Lluís Companys 23, 08010 Barcelona, Spain

*E-mail: fbaiutti@irec.cat; atarancon@irec.cat

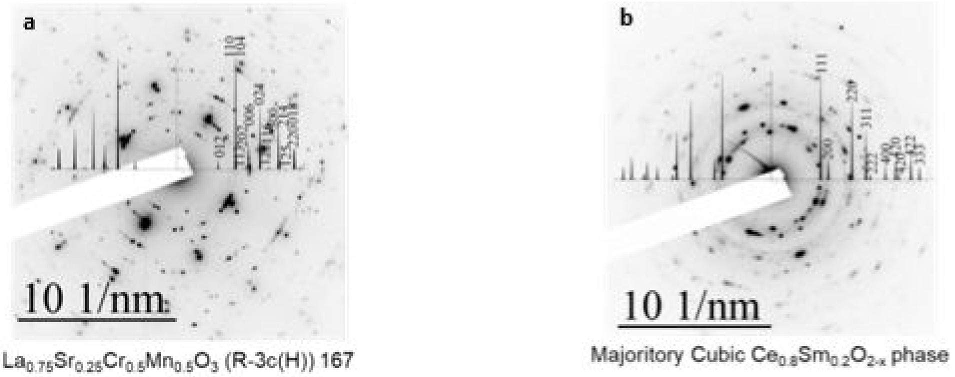

Figure S1. SAED patterns for the two heterostructures: (a) LSCrMn-SDC_{BL} and (b) LSCrMn-SDC_{NC}

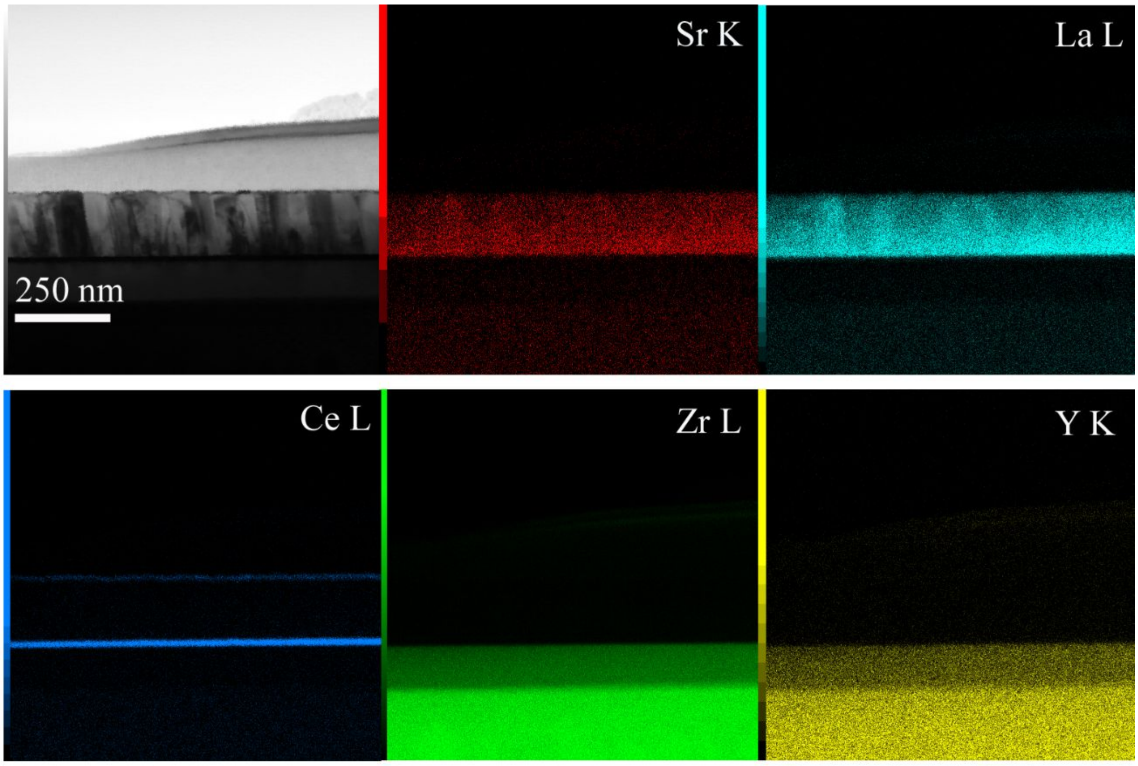

Figure S2. EDX elemental distribution maps of LSCrMn-SDC$_{BL}$ calculated using the Sr-K, La-L, Ce-L, Zr-L and Y-K and signals.

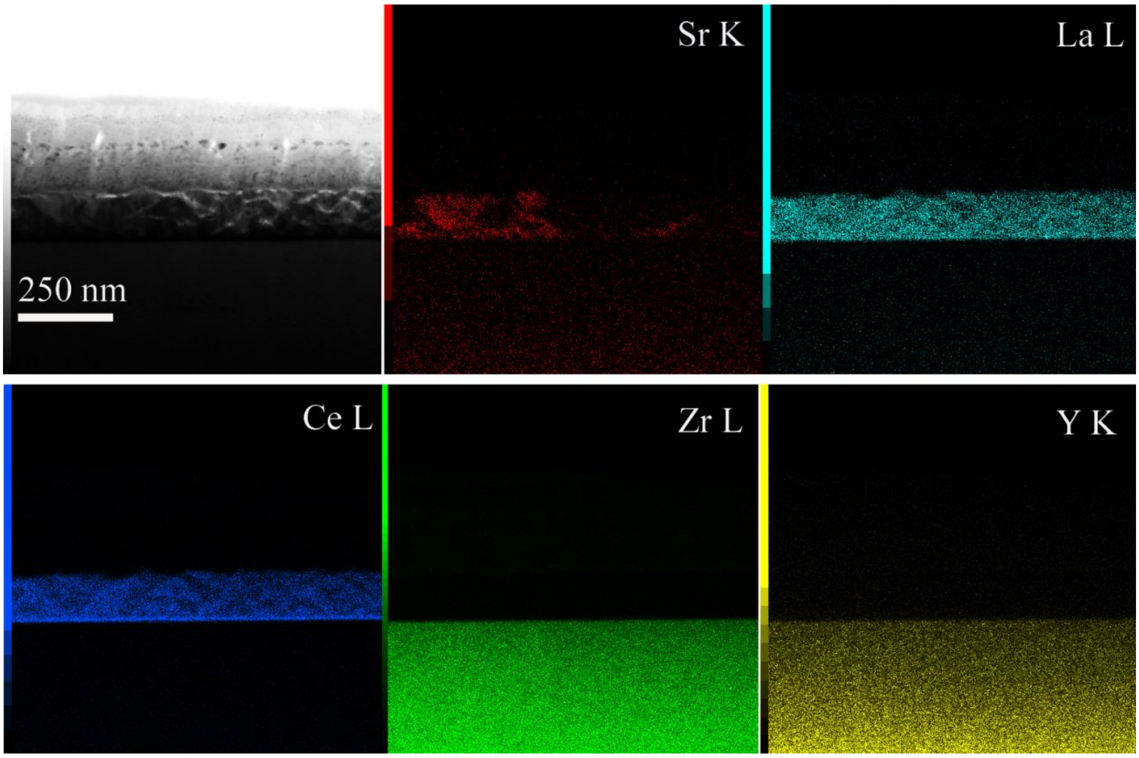

Figure S3. EDX elemental distribution maps of LSCrMn-SDC$_{NC}$ calculated using the Sr-K, La-L, Ce-L, Zr-L and Y-K and signals.

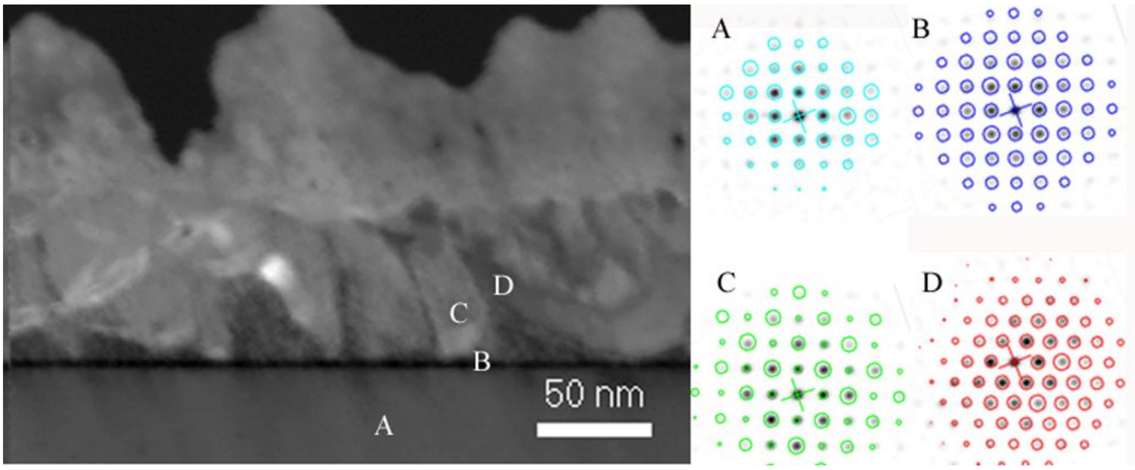

Figure S4. LSCrMn-SDC$_{NC}$ phase identification by ASTAR. Superposition of the electron diffraction patterns of selected regions (A to D) with the calculated matching phases for: YSZ Fm3m 225 (A), CGO Fm3m 225 (B), SDC Fm3m 225(C), LSCrMn R-3c(H) 167 (D).

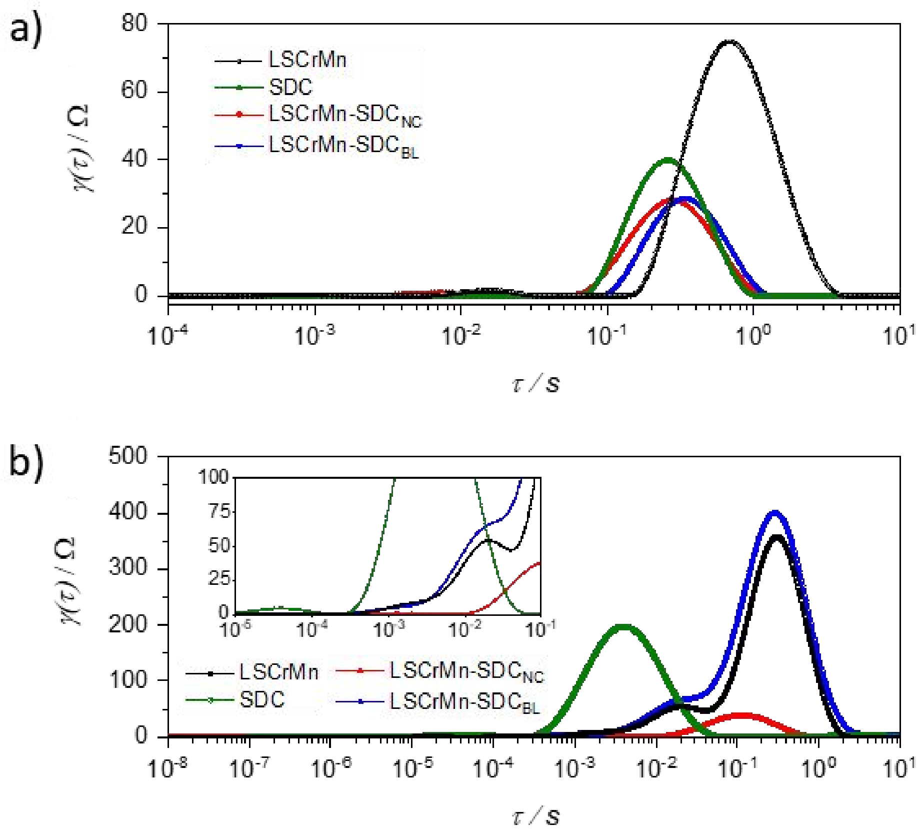

Figure S5. DRT analysis of the measurements carried out under (a) wet hydrogen and (b) synthetic air atmosphere at 750 °C.

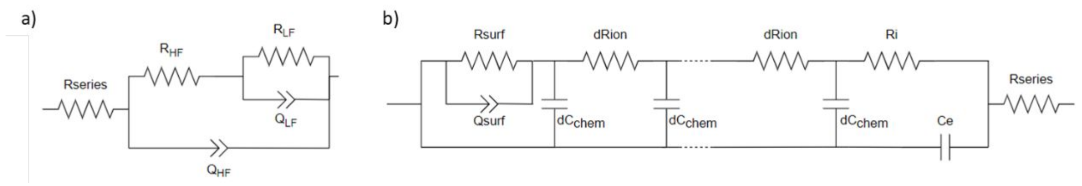

Figure S6. Equivalent circuits used for fitting the impedance spectra: (a) Circuit in conditions of high ionic conduction simplified from (b) a modified Jamnik-Maier equivalent circuit.

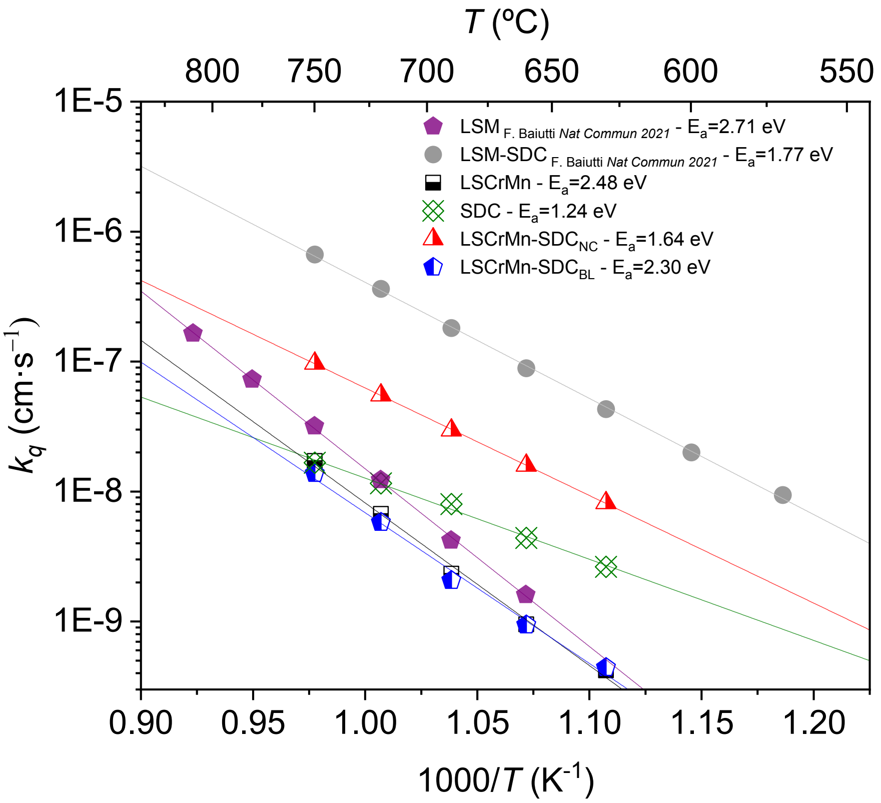

Figure S7. Oxygen surface exchange coefficient k_q evolution with temperature for all the materials studied.

Supplementary Note 1

True capacitances calculated from the high and low frequency arcs are collected in **Table S1.** In the case of the high frequency process, capacitances in the order of 10^{-4}-10^{-5} F/cm^2 are found, which are representative of diffusion processes[1] or of the double capacitance at the electrolyte/electrode interface[2]. The high frequency contribution can therefore be related to oxygen diffusion along the material or across the interface electrolyte/electrode[1,3]. The low frequency arc is instead associated to the surface

reactions, in agreement with previous works.[1,4–7] This interpretation is also supported by the DRT analysis presented in **Figure S6a**, which shows two main contributions for the impedance: a minor contribution at lower characteristic times (10^{-2}-10^{-3} s), and a major peak in the higher region (1-10^{-1} s). The capacitance associated to the low frequency arc is attributed the sum of the surface process and of the chemical capacitance C_{chem} of the films, which is expected to be the predominant in systems involving mixed conductors[8], and giving rise to values in the order of $\approx 10^3$ F/cm^3. These values are in agreement with what has also been reported in other studies involving perovskite and ceria-based MIECs[9–11].

Table S1. Capacitance values for the high and low frequency arcs shown in **Error! Reference source not found.**a.

Material	C (F/cm^2)	C_{chem} (F/cm^3)	n	
	High Frequency	Low Frequency	High Frequency	Low Frequency
LSCrMn	2.22 x 10^{-4}	1190.79	0.81	0.91
LSCrMn-SDC_{NC}	7.98 x 10^{-5}	1281.59	0.68	0.91
LSCrMn-SDC_{BL}	1.83 x 10^{-4}	1917.25	0.82	0.96
SDC	3.19 x 10^{-4}	999.88	0.89	0.94

Supplementary Note 2

It has been discussed that the two materials with a continuous LSCrMn layer (i.e. single LSCrMn and LSCrMn-SDC_{BL}) also present a diffusion-limited process, hence their performance would be influenced by the thickness of the electrode. Nonetheless, the small thickness difference between the LSCrMn-based structures (≤10%) allows to make a comparative analysis in between the architectures without expecting a different effect on

the performance due to the film thickness. Moreover, as it can be clearly observed from the D_q and k_q values extracted and reported in Table S2 –for the measurements at 750 °C– and in Figure S7 for the evolution of k_q with temperature, both LSCrMn and LSCrMn-SDC_{BL} present the highest surface resistance contribution among the rest of the other two materials tested, hence the diffusion contribution does not hinder the conclusions extracted in the analysis. With regards to the SDC layer, it has been discussed that the measurements in both conditions –reducing and oxidizing– are limited by the reaction in the surface, with no significant mass-transport contribution in the bulk. This is clearly observed when comparing the diffusivity values reported in Table S2, which shows a variation of six orders of magnitude between LSCrMn-based materials and SDC. Moreover, if we take the oxygen diffusivity of SDC (taken from the calculations reported in Vives et al. Ceram. Int. 2019 at 700 °C[12]) and consider a thickness of 150 nm (i.e. a value comparable to the thickness of the LSCrMn-based materials analyzed), a simple calculation would lead to a resistance of ≈3 mΩ.cm^2 at 700 °C, which would be even lower when extrapolated to the 750 °C that we have considered as reference values. Hence, based on this analysis a direct comparison between a thinner SDC and thicker LSCrMn layers is suitable for drawing conclusions on the electrochemical activity upon the surface reaction.

Table S2. Mass-transport parameters extracted from the EIS measurements obtained in air at 750 ºC. The SDC oxygen diffusivity value reported has been taken from Vives et al.[12] for comparison with the values calculated in this work.

Material	D_q (cm^2/s)	k_q (cm/s)
LSCrMn	4.95E-13	1.72E-08
LSCrMn-SDC_{BL}	3.26E-13	1.38E-08
LSCrMn-SDC_{NC}	-	9.67E-08
SDC	1.27E-07*	1.67E-08
*Extracted from Vives et al Ceram. Int. 2019 at 700 °C		

References

(1) Primdahl, S.; Liu, Y. L. Ni Catalyst for Hydrogen Conversion in Gadolinia-Doped Ceria Anodes for Solid Oxide Fuel Cells. *J. Electrochem. Soc.* **2002**, *149* (11), A1466–A1472. https://doi.org/10.1149/1.1514234.

(2) Holtappels, P.; Vinke, I. C.; de Haart, L. G. J.; Stimming, U. Reaction of Hydrogen/Water Mixtures on Nickel-Zirconia Cermet Electrodes: II. AC Polarization Characteristics. *J. Electrochem. Soc.* **1999**, *146* (8), 2976–2982. https://doi.org/10.1149/1.1392038.

(3) Murray, E. P.; Barnett, S. A. (La,Sr)MnO3-(Ce,Gd)O2-x Composite Cathodes for Solid Oxide Fuel Cells. *Solid State Ion.* **2001**, *143* (3–4), 265–273. https://doi.org/10.1016/S0167-2738(01)00871-2.

(4) Burnat, D.; Nasdaurk, G.; Holzer, L.; Kopecki, M.; Heel, A. Lanthanum Doped Strontium Titanate - Ceria Anodes: Deconvolution of Impedance Spectra and Relationship with Composition and Microstructure. *J. Power Sources* **2018**, *385* (February), 62–75. https://doi.org/10.1016/j.jpowsour.2018.03.024.

(5) Park, J. H.; Lee, J.-H.; Yoon, K. J.; Kim, H.; Ji, H.-I.; Yang, S.; Park, S.; Han, S. M.; Son, J.-W. A Nanoarchitectured Cermet Composite with Extremely Low Ni Content for Stable High-Performance Solid Oxide Fuel Cells. *Acta Mater.* **2021**, *206*, 116580. https://doi.org/10.1016/j.actamat.2020.116580.

(6) Jung, W.; Gu, K. L.; Choi, Y.; Haile, S. M. Robust Nanostructures with Exceptionally High Electrochemical Reaction Activity for High Temperature Fuel Cell Electrodes. *Energy Environ. Sci.* **2014**, *7* (5), 1685–1692. https://doi.org/10.1039/c3ee43546f.

(7) Nakamura, T.; Kobayashi, T.; Yashiro, K.; Kaimai, A.; Otake, T.; Sato, K.; Mizusaki, J.; Kawada, T. Electrochemical Behaviors of Mixed Conducting Oxide Anodes for Solid Oxide Fuel Cell. *J. Electrochem. Soc.* **2008**, *155* (6), B563–B569. https://doi.org/10.1149/1.2901047.

(8) Jamnik, J.; Maier, J. Generalised Equivalent Circuits for Mass and Charge Transport: Chemical Capacitance and Its Implications. *Phys. Chem. Chem. Phys.* **2001**, *3* (9), 1668–1678. https://doi.org/10.1039/b100180i.

(9) Chueh, W. C.; Haile, S. M. Electrochemical Studies of Capacitance in Cerium Oxide Thin Films and Its Relationship to Anionic and Electronic Defect Densities. *Phys. Chem. Chem. Phys.* **2009**, *11* (37), 8144–8148. https://doi.org/10.1039/b910903j.

(10) Lai, W.; Haile, S. M. Impedance Spectroscopy as a Tool for Chemical and Electrochemical Analysis of Mixed Conductors: A Case Study of Ceria. *J. Am. Ceram. Soc.* **2005**, *88* (11), 2979–2997. https://doi.org/10.1111/j.1551-2916.2005.00740.x.

(11) Rupp, G. M.; Limbeck, A.; Kubicek, M.; Penn, A.; Stöger-Pollach, M.; Friedbacher, G.; Fleig, J. Correlating Surface Cation Composition and Thin Film Microstructure with the Electrochemical Performance of Lanthanum Strontium Cobaltite (LSC) Electrodes. *J Mater Chem A* **2014**, *2* (19), 7099–7108. https://doi.org/10.1039/C3TA15327D.

(12) Vives, S.; Ramel, D.; Meunier, C. Molecular Dynamics Study in the Ce0.9M0.1O1.95 (M=Gd, Sm) Doped and Co-Doped CeO2 Systems: Structure and Oxygen Diffusion. *Ceram. Int.* **2019**, *45* (17), 21625–21634. https://doi.org/10.1016/j.ceramint.2019.07.158.
